# Midnight punctual tachycardia

**DOI:** 10.1007/s12471-025-01959-y

**Published:** 2025-05-07

**Authors:** Robin Kuijpers, Kim Smulders, Pepijn van der Voort, René Tio, Luuk Otterspoor

**Affiliations:** Department of Cardiology, Catharina Hospital, Eindhoven, The Netherlands

## Answer

**Fig. 1:**
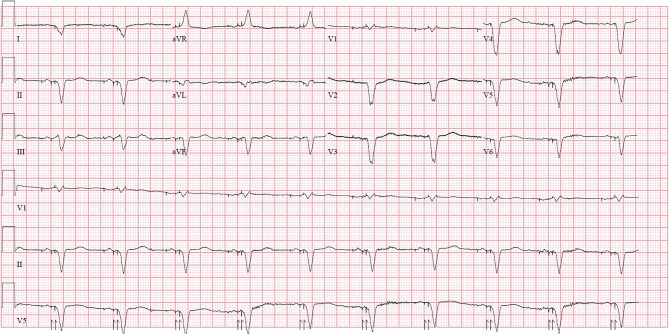
Resting ECG shows an atrial-sensed and biventricular-paced rhythm with a frequency of 60 beats per minute

The timing of the ventricular tachycardia (VT) onset is remarkable, occurring exactly at midnight, with the final episode each night at 2:30 a.m. The CRT‑D device is a Medtronic Crome, equipped with a conventional left ventricular lead via the coronary sinus, programmed in DDD mode with biventricular pacing, as illustrated in Fig. 1.

**Fig. 2:**
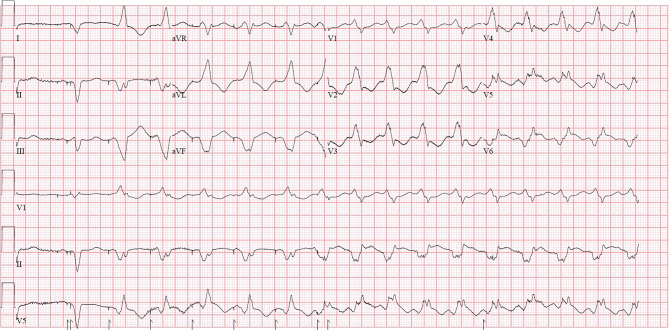
This ECG shows a biventricular-paced beat followed by five LV-only paced beats, after which ventricular tachycardia is initiated. The morphology of the VT closely resembles the LV-only paced beats

The capture management system tests the pacing threshold of each lead each night at midnight, beginning with the LV lead. With LV-only pacing, as illustrated in Fig. 2, after five paced complexes, VT is initiated. The VT morphology closely resembles the paced complex. Threshold testing fails due to VT and automatically restarts every 30 min, up to six attempts, explaining the recurrent arrhythmia each night.

In this patient, the LV lead tip was positioned within the inferoposterolateral scar, confirmed by voltage mapping. Scar tissue contains areas of slow conduction, and pacing near or within scar tissue increases repolarisation heterogeneity, heightening the risk of VT [1]. The incidence of VT initiation due to CRT‑D is approximately 4% [2].

Deactivation of the LV lead’s capture management system eliminated VT initiation, and with biventricular pacing, no further VT episodes occurred. Other solutions include relocating the LV lead or switching to left bundle branch pacing [3]. VT ablation was performed due to other VT morphologies, after which the patient remained free from ventricular arrhythmias.
